# Zeitlich begrenzter Therapieversuch („time-limited trial“, TLT) auf der Intensivstation: Handlungsempfehlung der Sektion Ethik der DIVI und der Sektion Ethik der DGIIN

**DOI:** 10.1007/s00063-024-01112-4

**Published:** 2024-02-12

**Authors:** Susanne Jöbges, Anna-Henrikje Seidlein, Kathrin Knochel, Andrej Michalsen, Gunnar Duttge, Alexander Supady, Jochen Dutzmann, Stefan Meier, Iris Barndt, Gerald Neitzke, Friedemann Nauck, Annette Rogge, Uwe Janssens

**Affiliations:** 1 Klinik für Anästhesiologie und Intensivmedizin, Charité-Universitätsmedizin Berlin, Campus Virchow-Klinikum, Augustenburger Platz 1, 13353 Berlin, Deutschland; 2 Institut für Ethik und Geschichte der Medizin, Universitätsmedizin Greifswald, Greifswald, Deutschland; 3 Institut für Geschichte und Ethik der Medizin, Technische Universität München, München, Deutschland; 4 Klinik für Anästhesiologie, Intensivmedizin, Notfallmedizin und Schmerztherapie, Klinikum Konstanz, Konstanz, Deutschland; 5 Institut für Kriminalwissenschaften, Abteilung für strafrechtliches Medizin- und Biorecht, Georg-August-Universität Göttingen, Göttingen, Deutschland; 6 Interdisziplinäre Medizinische Intensivtherapie, Universitätsklinikum Freiburg, Freiburg i.Br, Deutschland; 7 Klinik für Innere Medizin III, Universitätsmedizin Halle (Saale), Halle (Saale), Deutschland; 8 Klinik für Anästhesiologie, Universitätsklinikum Düsseldorf, Düsseldorf, Deutschland; 9 Klinik für Kardiologie und internistische Intensivmedizin, Universitätsklinikum Johannes Wesling, Minden, Deutschland; 10 Institut für Ethik, Geschichte und Philosophie der Medizin, Medizinische Hochschule Hannover, Hannover, Deutschland; 11 Klinik für Palliativmedizin, Universitätsmedizin Göttingen, Göttingen, Deutschland; 12 Fachbereich Neurologie, Paracelsus Nordseeklinik Helgoland, Helgoland, Deutschland; 13 Klinik für Innere Medizin und Internistische Intensivmedizin, St.-Antonius-Hospital, Eschweiler, Deutschland

**Keywords:** Intensivmedizin, Medizinethik, Protokolle und Leitlinien, Qualität in der Gesundheitsversorgung, Prognose Behandlungsergebnis, Critical care, Medical ethics, Protocols and guidelines, Quality in health care, Prognosis treatment outcome

## Abstract

Die Zunahme der intensivmedizinischen Behandlungsverfahren ist mit einer Steigerung der Komplexität der Entscheidungen über Auswahl, Anwendung und Dauer der Therapiemaßnahmen verbunden. Ob im individuellen Fall ein Behandlungsziel erstrebenswert und der dafür notwendige Behandlungsweg akzeptabel ist, hängt von den persönlichen Wünschen, Werthaltungen und Lebensentwürfen der Patientinnen und Patienten ab. Häufig bestehen Unsicherheiten darüber, ob ein patientenzentriertes Behandlungsziel erreicht werden kann. Der Einsatz eines zeitlich begrenzten Therapieversuches (TLT) als eine verbindliche Übereinkunft zwischen dem Behandlungsteam und der Patientin/dem Patienten bzw. dem juristischen Stellvertretenden zu einem Behandlungskonzept über einen definierten Zeitraum auf der Intensivstation kann hilfreich sein, um Unsicherheiten zu reduzieren und die Fortführung intensivmedizinischer Maßnahmen im Sinne des/der Patient/in besser einordnen und rechtfertigen zu können.

**Zusatzmaterial online:**

Die Online-Version dieses Beitrags (10.1007/s00063-024-01112-4) enthält 2 Fallbeispiele aus der Chirurgie und der Inneren Medizin zur Durchführung eines TLT.

## Abstract (en)

The rise in intensive care treatment procedures is accompanied by an increase in the complexity of decisions regarding the selection, administration and duration of treatment measures. Whether a treatment goal is desirable in an individual case and the treatment plan required to achieve it is acceptable for the patient depends on the patient’s preferences, values and life plans. There is often uncertainty as to whether a patient-centered treatment goal can be achieved. The use of a time-limited treatment trial (TLT) as a binding agreement between the intensive care unit (ICU) team and the patient or their legal representative on a treatment concept over a defined period of time in the ICU can be helpful to reduce uncertainties and to ensure the continuation of intensive care measures in the patients’ best interest.

## Infobox 1 Weitere beteiligte Mitglieder der Sektionen Ethik der DIVI und der DGIIN

Hilmar Burchardi (Göttingen), Friedrich Ebinger (Paderborn), Tizia Johanna Eggardt (Berlin), Steffen Grauthoff (Herford), Peter Gretenkort (Krefeld), Christiane Hartog (Berlin), Stefan Kleinschmidt (Homburg (Saar)), Maike Lyall (Berlin), Urs Münch (Berlin), Friedemann Nauck (Göttingen), Brigitte Parvu (Zweibrücken), Raffael Riegel (Bonn), Fred Salomon (Lemgo), Ralf Schumacher (Mettmann), Torsten Schröder (Berlin), Herwig Stopfkuchen (Mainz), Esther Tautz (Freiburg), Claudia Weber (Hamburg), René Wildenauer (Wiesentheid).

## 1. Einleitung

Die kontinuierliche Weiterentwicklung medikamentöser und apparativer intensivmedizinscher Verfahren führt zu einer stetigen Zunahme an Behandlungsmöglichkeiten. So sind für einige Patientinnen und Patienten heute lebensverlängernde intensivtherapeutische Maßnahmen möglich, die vor einigen Jahren noch nicht gegeben waren [19]. Damit verbunden ist eine zunehmende Komplexität der Entscheidungen über Auswahl, Anwendung und Dauer der Therapiemaßnahmen. Daraus entstehen Belastungen sowohl für Patientinnen und Patienten sowie ihre Zugehörigen als auch für das behandelnde medizinische Personal, ohne dass immer klar ist, ob das von der Patientin oder vom Patienten gewünschte Therapieziel tatsächlich erreicht werden kann.

Entscheidungen über den Beginn, die Fortführung und die Beendigung intensivmedizinischer Maßnahmen erfordern immer eine Einschätzung ihrer Sinnhaftigkeit und Angemessenheit [9]. Diese Bewertung ist einerseits Teil der Indikationsstellung und andererseits Ausdruck des (mutmaßlichen) Patientenwillens. Ob im individuellen Fall ein Behandlungsziel erstrebenswert und der dafür notwendige Behandlungsweg akzeptabel ist, hängt von den persönlichen Wünschen, Werthaltungen und Lebensentwürfen der Patientinnen und Patienten ab. In der intensivmedizinischen Praxis bestehen oft Unsicherheiten über das Therapieziel, den Therapieverlauf und die Prognose [10, 26]. Daraus resultiert eine zusätzliche Verantwortung für das Behandlungsteam. Eine mögliche Strategie im Umgang mit diesen Unsicherheiten ist die Durchführung eines zeitlich begrenzten Therapieversuches („time-limited trial“, TLT). Empirische Studien zum TLT zeigen Vorteile und positive Auswirkungen für Teamentscheidungen und ein patientenzentriertes Behandlungsergebnis [4, 25, 26].

Die vorliegende Handlungsempfehlung umfasst Definition, ethische Argumente, empirische Daten und Empfehlungen für die Umsetzung eines TLT in der Praxis.

## 2. Definition und Präzisierung: TLT als Werkzeug

Ein TLT kann bei bestehenden Zweifeln an Sinnhaftigkeit und Angemessenheit intensivmedizinischer Maßnahmen vereinbart werden. Das TLT umfasst in dieser Situation eine *verbindliche Übereinkunft zwischen dem Behandlungsteam und der Patientin oder dem Patienten* bzw. dem juristischen Stellvertreter zu einem Behandlungskonzept *über einen definierten Zeitraum *[14, 20].

### Zeitpunkt für einen TLT

Zum Zeitpunkt der Übereinkunft für einen TLT wird eine Behandlung noch nicht als sicher aussichtlos eingeschätzt. Es besteht aber bereits eine hohe Unsicherheit, ob mit der Fortführung der Behandlung das bisherige Therapieziel im Einklang mit dem (mutmaßlichen) Patientenwillen erreicht werden kann [26]. Ein *TLT *kann in dieser Situation *als ein letzter Behandlungsversuch* bei äußerst kritischer Prognose eingesetzt werden.

Das TLT ist *kein* Instrument für folgende Situationen:

- Die Aussichtslosigkeit ist festgestellt, aber die Therapiezieländerung wird von der Patientin oder dem Patienten bzw. dem gesetzlichen Stellvertreter abgelehnt: Eine Änderung des Therapieziels und das Zulassen des Sterbens muss in diesem Fall wegen fehlender oder nicht mehr gegebener Indikation erfolgen [6].
- Der Patientenwille hinsichtlich der aktuell durchgeführten Behandlungsmaßnahmen ist unklar: Der (meist mutmaßliche) Patientenwille muss immer schnellstmöglich geklärt werden.
- Die Maßnahme ist klar indiziert und entspricht, soweit beurteilbar, dem (mutmaßlichen) Patientenwillen. Es wird auf den Behandlungserfolg gewartet: Die Behandlung erfolgt zunächst ohne Fristsetzung.

### Begründung für den Einsatz eines TLT

Bei prolongierten Krankheitsverläufen auf der Intensivstation ist die Prognostizierung mit Unsicherheiten behaftet, die das Behandlungsteam anerkennen und kommunizieren muss. Ein TLT ist gerechtfertigt, wenn damit eine größere Prognosesicherheit erreicht werden kann [4, 10]. Auf dieser Grundlage kann außerdem einem möglicherweise geschärften oder neu formulierten (mutmaßlichen) Patientenwillen Rechnung getragen werden. So können Vertrauen und Verlässlichkeit für das weitere Vorgehen gestärkt werden.

Die Entscheidung für ein TLT verhindert damit sowohl, dass die Therapie zu früh begrenzt oder beendet als auch dass sie unkritisch fortgesetzt wird. Durch eine zu frühe Therapiebegrenzung könnte der bleibende Eindruck entstehen, die Patientin oder der Patient hätte noch eine Überlebenschance gehabt. Verbindliche Absprachen über ein TLT verhindern zugleich, dass die Therapie ohne Reflexion und ggf. perspektivlos fortgesetzt wird [2, 12, 13]. Beides belastet nicht nur die Patientinnen und Patienten, sondern auch das Behandlungsteam und die An- und Zugehörigen [1, 23, 26].

Ein TLT kann aus ethischer Perspektive den bestmöglichen Kompromiss zwischen einem gebotenen und gerade noch vertretbaren Behandlungsversuch („Leben retten“) und einer gleichermaßen gebotenen palliativen Begleitung („keine Überversorgung am Lebensende“) darstellen. Eine Überversorgung kann ebenso wie eine ethisch nicht gerechtfertigte Sterbeverlängerung *„moral distress“* und Burn-out im Behandlungsteam verursachen oder verstärken [16].

## 3. Durchführung

Ein TLT kann zu verschiedenen Zeitpunkten im Verlauf der Behandlung vereinbart werden:

- bei Krankenhausaufnahme in der zentralen Notaufnahme;
- bei angefragter Aufnahme auf die Intensivstation, z. B. von der Allgemeinstation, sowie
- im weiteren Verlauf des Intensivaufenthalts.

Die Entscheidung, ein TLT anzubieten, sollte in jeder dieser Situationen interdisziplinär und interprofessionell unter Leitung einer erfahrenen Intensivmedizinerin oder eines erfahrenen Intensivmediziners im Teamkonsens getroffen werden. Für die Moderation von interdisziplinären oder interprofessionellen Konflikten kann ein ethisches Beratungsgremium zugezogen werden. Gerade prognostische Unsicherheit ist ein häufiger Konsultationsgrund und kann in einer Ethikberatung spezifisch thematisiert werden [6, 22].

### Klärung der Behandlungsoptionen

In einer Behandlungssituation, die nahezu aussichtslos ist, werden Therapieoptionen mit konkreten Erfolgskriterien und festgelegter Dauer vereinbart, die dem gegenwärtigen Therapieziel der Patientin oder des Patienten entsprechen [7, 8]. Bereits festgelegte Vereinbarungen zur Therapielimitierung werden beibehalten oder erweitert, wenn dies angezeigt ist.

### Zielvereinbarungen eines TLT

Bei der Planung eines TLT werden *patientenindividuelle, überprüfbare* Kriterien festgelegt [8, 20]. Diese sollen eine Zustandsänderung der Patientin oder des Patienten im Behandlungsverlauf transparent und für alle Beteiligten nachvollziehbar objektivieren. Scores oder einzelne Parameter sind hier im Regelfall nicht hilfreich, da diese kein patientenindividuelles Outcome abbilden. Insbesondere besteht die Gefahr, sich auf Einzelbefunde oder minimale Änderungen zu fokussieren und das übergeordnete Therapieziel aus den Augen zu verlieren. Die Kriterien eines TLT müssen insgesamt objektivierbar und reproduzierbar sein und definieren den Erfolg des TLT.

### Dauer eines TLT

Soweit möglich sollte für die Dauer eines TLT ein Zeitraum in Betracht gezogen werden, der unter Fortsetzung der lebensverlängernden Maßnahmen dem erfahrungsgemäß erforderlichen Zeitraum bis zu einer erwartbaren Verbesserung entspricht. Üblicherweise werden dafür wenige Tage angenommen [8, 15, 20]. Hierbei ist zu beachten, dass sowohl eine zu kurze als auch eine zu lange Dauer des TLT der Patientin oder dem Patienten schaden können [26].

### Evaluation eines TLT

Die tägliche Reevaluation von Therapiemaßnahmen erfolgt mit besonderem Blick auf die prognostische Unsicherheit und die getroffenen Vereinbarungen eines TLT [26]:

- Zeigt sich eine klinische Verbesserung der Erkrankung, wird eine Fortführung der Therapie mit dem bisherigen Therapieziel festgelegt.
- Eine deutliche klinische Verschlechterung während des laufenden TLT bedingt in der Regel eine Therapiezieländerung, gegebenenfalls auch vor Ende des vereinbarten Zeitraums.
- Zeigt sich bis zum Ende des vereinbarten Zeitraums keine Verbesserung nach den zuvor bestimmten Kriterien – wobei eine klinisch unveränderte Situation wie eine Verschlechterung zu bewerten ist, erfolgt gemäß der Vereinbarung eine Therapiezieländerung. Bei fortbestehender erheblicher prognostischer Unsicherheit kann *ausnahmsweise* ein erneutes TLT vereinbart werden [20]. Dies muss allerdings ärztlicherseits sehr gut begründet werden und darf keinesfalls Ausdruck einer Entschlussträgheit im Behandlungsteam sein.

## 4. Kommunikation

Eine transparente und umfassende Kommunikation bei der Vereinbarung eines TLT beinhaltet die Erörterung der vorliegenden Behandlungssituation mit ihren möglichen Optionen [4]. Die Sorge, dass die Behandlung bereits aussichtslos geworden ist, sollte genauso geäußert werden wie die, dass noch mögliche Behandlungschancen nicht wahrgenommen werden [21]. Klare Vereinbarungen, welche Konsequenzen aus dem Verlauf des TLT gezogen werden, sind unverzichtbar [8]. Bei der Kommunikation über die Durchführung eines TLT muss darauf geachtet werden, dass allen Beteiligten, insbesondere den medizinischen Laien, die Bedeutung der Begriffe „prognostische Unsicherheit“, „Therapieoptionen“ und der Erfolgs- und Misserfolgskriterien deutlich wird (*Infobox **2*). Dies ist insbesondere deshalb notwendig, weil auch scheinbar einfache Begriffe, wie z. B. „Behandelbarkeit“, sehr unterschiedlich wahrgenommen werden können und gegebenenfalls unrealistische Hoffnungen wecken [3].

In allen Gesprächen sollte deshalb auch bedacht werden, dass die individuelle Hoffnung davon abhängt, ob ein positiver Ausgang für möglich gehalten wird, und nicht so sehr davon, ob er statistisch wahrscheinlich ist [17]. Es ist unmöglich und auch entbehrlich, Hoffnung auf Besserung argumentativ zu widerlegen. In der Kommunikation sowohl im Team als auch mit Angehörigen muss darauf geachtet werden, dass sich alle wahrgenommen und mitgenommen fühlen. Wenn die Kommunikation über Hoffnung und Wahrscheinlichkeit gelingt, dann ist die Vereinbarung eines TLT über die Grenzen der unterschiedlichen Wahrnehmungen hinaus möglich.

Ein TLT ist ein Behandlungsversuch, der auch Raum bietet, um parallel ein mögliches Sterben anzusprechen, auch dann, wenn noch Überlebenschancen bestehen. Die Kommunikation mit den Patientinnen und Patienten bzw. Zugehörigen soll genutzt werden, um rechtzeitige multiprofessionelle Palliativversorgung zu thematisieren und zu integrieren [5].

### Infobox 2 Möglichkeiten, eine unsichere Prognose zu verbalisieren

- „Wenn es während des vereinbarten Zeitraums keine Verbesserung des Gesundheitszustands gibt, sind wir sicher, dass eine Fortsetzung aussichtslos geworden ist.“
- „Wir unternehmen diese Schritte, damit wir alle sehen, dass wir alles uns Mögliche für ihn/sie getan haben.“
- „Wir haben alles uns Mögliche getan, allerdings hat sich der körperliche Zustand bisher nicht verbessert. Es ist daher notwendig, die lebensverlängernden Maßnahmen nach der vereinbarten Zeit zu beenden. Sonst schaden wir ihm/ihr nur.“
- „Wenn sich während des vereinbarten Zeitraums nichts verbessert, sind wir sehr sicher: Wir sind außerstande, den Gesundheitszustand und die Lebensqualität zu erreichen, die Ihre Angehörige/Ihr Angehöriger sich wünscht.“
- „Wir können Ihnen keine Garantie geben, dass die Therapie im vereinbarten Zeitraum zum gewünschten Erfolg führt. Daher scheint es sinnvoll, parallel auch eine palliative Symptombehandlung mitzudenken, um Leid zu lindern und Lebensqualität in jedem Lebensabschnitt, auch im Sterbeprozess, zu ermöglichen.“

## 5. Dokumentation

Die Dokumentation der TLT-Modalitäten soll strukturiert erfolgen. Dies kann in bereits existierende Dokumentationsbögen oder -formen integriert werden (z. B. strukturiertes Angehörigengespräch, Therapiebegrenzungsbogen [18]). Auch eine davon getrennte Dokumentation kann klinikintern vereinbart werden. Es sollten mindestens folgende Punkte dokumentiert werden (*Infobox **3*):

### Infobox 3 Dokumentation eines TLT

- Knappe Beschreibung der individuellen Ausgangssituation, Anlass, Beteiligte (interdisziplinär, interprofessionell, patientenseitig)
- Begründung für das TLT (ggf. Verweis auf ethische Fallbesprechung)
- Festlegen der *Kriterien*, an denen der Erfolg/Misserfolg des TLT im individuellen Fall bemessen wird
- Dauer des TLT
- Festlegen des Zeitpunkts für ein erneutes strukturiertes Angehörigengespräch
- Behandlungsoptionen, geltende Therapiebegrenzungen, Einbindung von palliativmedizinischen Teams
- Tägliche Reevaluation der Situation
- Entscheidung im Verlauf oder zum vereinbarten Ende des TLT im Teamkonsens

## 6. Grenzen und Herausforderungen eines TLT

Ein TLT kann Hoffnungen wecken, die eine Therapiezieländerung möglicherweise erschweren können. Eine prognostische Restunsicherheit wird zudem auch mit dem TLT nicht gänzlich aufzulösen sein [24]. Die Frage nach dem „richtigen“ Zeitpunkt für eine Therapiezieländerung kann auch durch ein TLT nicht abschließend beantwortet werden. Wie viel Sicherheit es für die Rechtfertigung eines Therapiebeginns und einer Therapiezieländerung bedarf, bleibt davon unberührt und muss von den behandelnden Teams als empirisch belastbar (evidenzinformiert) und vertretbar eingeschätzt werden. Das bedeutet, dass nach wie vor subjektive Einschätzungen eine Rolle spielen, was Konfliktpotenzial bieten kann.

Die Durchführung eines TLT setzt bei allen Beteiligten die Bereitschaft voraus, entsprechend den Vereinbarungen zum TLT das Therapieziel zu ändern, lebensverlängernde Behandlungsmaßnahmen zu beenden und eine Palliativversorgung rechtzeitig in die Wege zu leiten. Falls Konflikte verbleiben, weil die Patientinnen oder Patienten, die An- und Zugehörigen oder die gesetzlichen Vertreter die medizinische Bewertung des Behandlungsteams nicht akzeptieren können oder weil es innerhalb des Behandlungsteams zu divergierenden Einschätzungen kommt, sollte ein ethisches Beratungsgremium hinzugezogen werden.

Besondere Herausforderungen in der Prognosestellung sind akute Erkrankungen des Gehirns, bei denen für längere Zeit unsicher ist, ob die Patientin oder der Patient das Bewusstsein wiedererlangen wird. Für diese Patientengruppe ist die Möglichkeit eines *längerfristigen *TLT in Betracht zu ziehen. Da die beschriebenen akuten zerebralen Ereignisse nach Überleben der Akutphase in einer jahrzehntelangen chronischen Bewusstseinsstörung münden können, ist eine im Rahmen des TLT vereinbarte Reevaluation von besonderer Relevanz [11].

## 7. Fazit

Behandlungsentscheidungen auf Intensivstationen unterliegen immer einer prognostischen Unsicherheit. Ein TLT ist ethisch gerechtfertigt, wenn das Erreichen des Therapieziels sehr unwahrscheinlich, aber noch nicht aussichtslos ist. Die Durchführung eines TLT setzt die Bereitschaft voraus, entsprechend den Vereinbarungen das Therapieziel zu ändern. Die Ausgangssituation, die Durchführung und die Folgen eines TLT zu kommunizieren, ist herausfordernd. Ein TLT kann Verlässlichkeit und Vertrauen stärken, Unsicherheit reduzieren, Überversorgung vermeiden und eine patientenzentrierte Intensivmedizin fördern.

### Supplementary Information




