# Supplementary material for: Time-limited trials (TLT) in the intensive care unit: Recommendations from the ethics section of the DIVI and the ethics section of the DGIIN
Source: Med Klin Intensivmed Notfmed. 2024 Feb 12;119(4):291–5. [Article in German] doi: 10.1007/s00063-024-01112-4 (PMC11059002; doi:10.1007/s00063-024-01112-4)
Supplement: Supplementary file 1 [file 63_2024_1112_MOESM1_ESM.pdf]

TLT -Fallbeispiel Chirurgie

**Knappe Beschreibung der individuellen Ausgangssituation,** Anlass, Beteiligte (interdisziplinär, interprofessionell, patientenseitig)	Patientin mit nekrotisierender Pankreatitis, multiple bislang kompensierte kardiale Vorerkrankungen<br>Zwei Monate intensivmedizinische Behandlung mit rezidivierenden septischen Schüben<br>Phasen der TAA, Kardioversion, Implantation eines passageren externen Schrittmachers<br>Prolongiertes Beatmungsweaning – Tracheotomie<br>Offene Bauchbehandlung bei multiplen Darm-, Pankreas-Fisteln, Parenterale Ernährung,<br>nach Etablierung eines stabilen Fistelsystems wäre nach erfolgter Genesung ein plastisch-rekonstruktiver Bauchdeckenverschluss notwendig<br>akut auf chronisches Nierenversagen – umtägige Dialyse<br>Erhöhte Transaminasen, Bilirubin 15mg/dl - undulierend, Gerinnung mäßig eingeschränkt<br>Thrombosen in V. jugularis interna beidseits, ZVK/Shaldon -Anlagen gestalten sich zunehmend schwierig<br>**Aktuell:** Erneuter septischer Schub mit weiteren Nekrosestraßen (CT), erneute Vigilanzminderung, erneute kardiale Dekompensation
**Begründung für das TLT,**<br>(ggf. Verweis auf ethische Fallbesprechung)	Im Gesamtkontext nahezu aussichtslose Situation
**Festlegen der Kriterien**, an denen der Erfolg/Misserfolg des TLT im individuellen Fall bemessen wird	kardiale Rekompensation, Katecholaminbedarf, Verlauf Infektparameter, Verlauf Leberwerte- Gerinnung, Laktatverlauf
**Dauer des TLT**	5 Tage
**Festlegen des Zeitpunktes für ein erneutes strukturiertes Angehörigengespräch**	In 5 Tagen
**Behandlungsoptionen**<br><br>**geltende Therapiebegrenzungen**,<br><br>**Einbindung von palliativmedizinischen Teams**	Da eine erneute interventionelle Drainage technisch unmöglich ist, nach kardialer Stabilisierung chirurgische Nekrosektomie als Ultima Ratio, ggf. Umstellung der Antibiose nach Befund, differenzierte Volumen- und Katecholamintherapie für den Zeitraum des TLT.<br><br>Keine ECMO, Keine CPR<br><br>Beginn palliativmedizinische Mitbehandlung
**Tägliche Re-Evaluation** der Situation	
**Entscheidung** im Verlauf oder zum vereinbarten Ende des TLT im Team-Konsens	**Verbesserung:** → Therapie fortführen<br>**Keine Änderung/Verschlechterung** der klinischen Situation: →Therapiezieländerung

Fallbeispiel Innere Medizin

**Knappe Beschreibung der individuellen Ausgangssituation,** Anlass, Beteiligte (interdisziplinär, interprofessionell, patientenseitig)	Patient mit Rezidiv einer malignen hämatologischen Erkrankung, Knochenkarzinose, palliative Situation mit unklarer Lebenszeit unter erneuter Chemotherapie<br>**Intensivstation:** Intubiert, invasiv beatmet, bei enoraler Blutung,<br>Sediert, presst, mehrfach kurze Reanimation im Rahmen von Hypoxiephasen<br>hochdosiert Katecholamine, Sepsis, ANV, Leberversagen<br>aktuell ist eine Chemotherapie als palliative Behandlung der Grunderkrankung nicht möglich<br><br>**aktuell:** erneute Sepsis bei Pneumonie, Verschlechterung der Beatmungssituation<br>Panzytopenie
**Begründung für das TLT**, (ggf. Verweis auf ethische Fallbesprechung)	Nahezu Aussichtslosigkeit auf Heilung<br>Eine Therapie der Grunderkrankung ist nicht möglich<br>ggf. falls Stabilisierung ist eine Chemotherapie möglich, aber weiterhin schlechte Prognose mit unklarer Lebenszeit, mit Risiko der erneuten Immunsuppression und Sepsis
**Festlegen der Kriterien,** an denen der Erfolg/Misserfolg des TLT im individuellen Fall bemessen wird	Verlauf:<br>- Katecholaminbedarf,<br>- Infektparameter,<br>- Leberwerte- Gerinnung,<br>- Laktatverlauf
**Dauer des TLT**	5 Tage
**Festlegen des Zeitpunktes für ein erneutes strukturiertes Angehörigengespräch**	In 5 Tagen
**Behandlungsoptionen**,<br><br><br><br><br><br>**geltende Therapiebegrenzungen,**<br><br>**Einbindung von palliativmedizinischen Teams**	Eskalation Antibiotikatherapie<br>Invasive Beatmung, Lagerung,<br><br>Transfusion,<br>Granulozyten stimulierender Faktor (GCSF)<br><br><br>keine ECMO, keine Reanimation<br><br>Beginn palliativmedizinische Mitbehandlung
**Tägliche Re-Evaluation** der Situation	
**Entscheidung** im Verlauf oder zum vereinbarten<br>Ende des TLT im Team-Konsens	**Verbesserung:** → Therapie fortführen<br>**Keine Änderung/Verschlechterung** der klinischen<br>Situation: →Therapiezieländerung

TAA: Tachyarrhythmia absoluta
CT: Computertomografie
ECMO: Extracorporale Membranoxygenierung
